# Traction and resection in treating gastric submucosal tumor growing extraluminally by using two snares like a ropeway

**DOI:** 10.1055/a-2081-8284

**Published:** 2023-05-26

**Authors:** Lei Gu, Yu Wu, Xiaowei Liu

**Affiliations:** 1 Department of Gastroenterology, Xiangya Hospital, Central South University, Changsha, Hunan, China; 2 Hunan International Scientific and Technological Cooperation Base of Artificial Intelligence Computer Aided Diagnosis and Treatment for Digestive Disease (2020CB1004), Changsha, Hunan, China; 3 National Clinical Research Center for Geriatric Disorders, Xiangya Hospital, Central South University, Changsha, Hunan, China

A 53-year-old woman with upper abdominal pain underwent computed tomography, which showed a lump with an extraluminal growth pattern located at the junction of the gastric body and antrum ( Fig. 1 ). Endoscopic full-thickness resection (EFTR) is a feasible approach for gastric submucosal tumor growing extraluminally 1 .

**Fig. 1:**
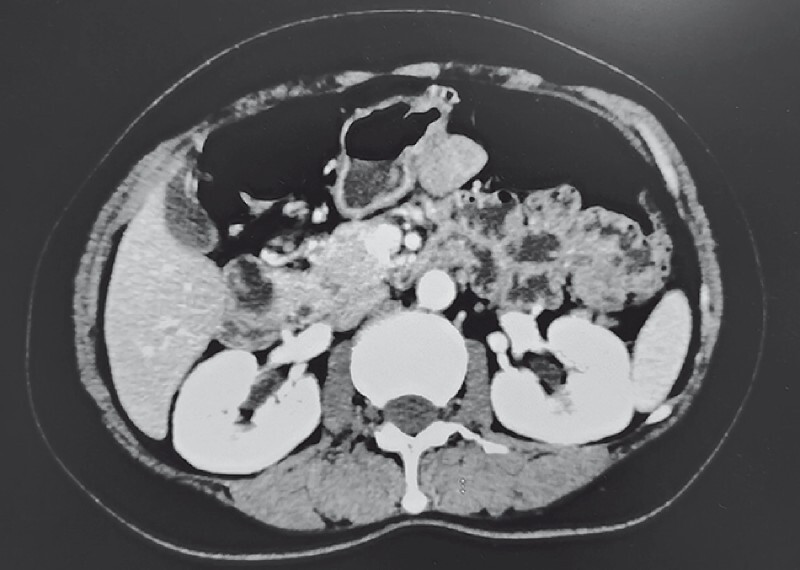
A submucosal tumor growing extraluminally was found at the junction of the gastric body and antrum.

After marking and submucosal injection, we incised the mucosa and submucosa along with the marker using a DualKnife (Olympus, Tokyo, Japan), and exposed part of the tumor. As we dissected along the margin of the tumor, we found that the tumor originated from the muscularis propria and mainly grew outside the stomach wall; thus we excised the muscularis propria layer near the tumor using an insulation-tipped knife. The major part of the tumor was in the abdominal cavity ( Fig. 2 a ). To expose the basal part and distal end of the lesion, and to prevent the tumor from falling into the abdominal cavity, a snare (SD-210U-25; Olympus) was inserted through the forceps channel to grasp the tumor and pull it into the stomach ( Fig. 2 b ). By using a pair of hemostatic forceps to keep the snare tight externally, we cut off the handle of the snare and pulled the endoscope out through the mouth. We then reinserted the endoscope with a second snare (through the forceps channel) into the stomach, with the second snare progressing over the line of the first snare like a ropeway ( Fig. 2 c , Video 1 ). Under the external traction of the first snare, we trapped and resected the junction of the tumor and gastric wall using the second snare. After retrieving the specimen ( Fig. 2 d ) by pulling back the first snare and endoscope, we reinserted the endoscope and sutured the wound using metal clips.

**Fig. 2:**
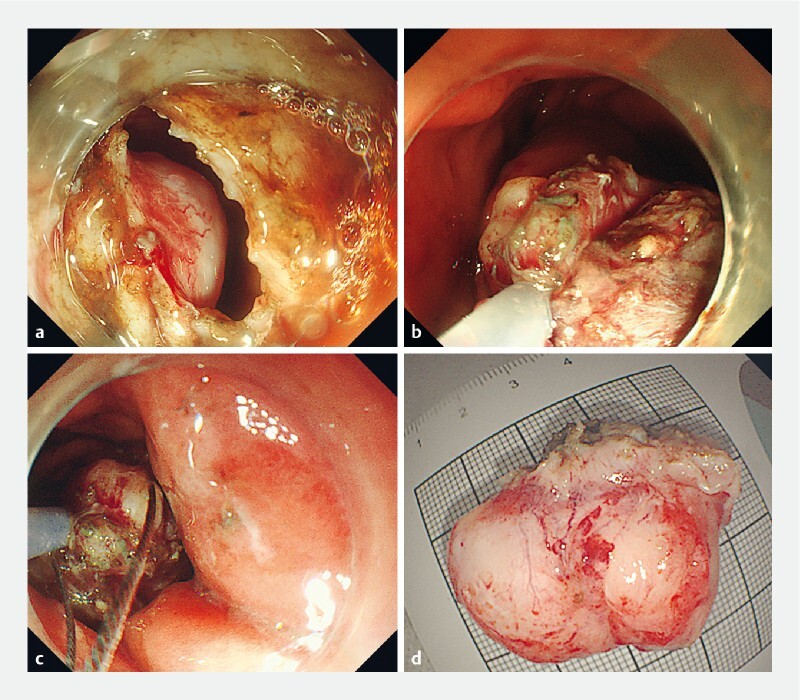
Endoscopic images. **a** The major part of the exposed tumor was located outside the gastric cavity. **b** The tumor was grasped and pulled into the gastric cavity by the first snare. **c** The endoscope with second snare was reinserted along the first snare. **d** The resected specimen was about 4.0 × 2.8 cm in size.

**Video 1** Endoscopic full-thickness resection of a gastric schwannoma growing extraluminally by using two snares like a ropeway.


The patient started a liquid diet and was discharged from hospital 2 days after the procedure. Postoperative pathological analysis revealed schwannoma. Ropeway-like application of two snares might simplified the procedure of EFTR for gastric submucosal tumors growing extraluminally ( Video 1 ).

Endoscopy_UCTN_Code_TTT_1AO_2AG

## Notes

Endoscopy E-Videos

https://eref.thieme.de/e-videos
*E-Videos* is an open access online section of the journal *Endoscopy* , reporting on interesting cases and new techniques in gastroenterological endoscopy. All papers include a high-quality video and are published with a Creative Commons CC-BY license. Endoscopy E-Videos qualify for HINARI discounts and waivers and eligibility is automatically checked during the submission process. We grant 100% waivers to articles whose corresponding authors are based in Group A countries and 50% waivers to those who are based in Group B countries as classified by Research4Life (see: https://www.research4life.org/access/eligibility/ ). This section has its own submission website at https://mc.manuscriptcentral.com/e-videos
